# New dates on dingo bones from Madura Cave provide oldest firm evidence for arrival of the species in Australia

**DOI:** 10.1038/s41598-018-28324-x

**Published:** 2018-07-19

**Authors:** Jane Balme, Sue O’Connor, Stewart Fallon

**Affiliations:** 1 Archaeology, School of Social Sciences, University of Western Australia, Crawley, 6009 Australia; 2 Department of Archaeology and Natural History, College of Asia and the Pacific, Australian National University, Canberra, ACT 0200 Australia; 3 Centre of Excellence for Australian Biodiversity and Heritage, Australian National University, Canberra, ACT 0200 Australia; 4 Research School of Earth Sciences, College of Science, Australian National University, Canberra, ACT 0200 Australia

## Abstract

The dingo is the only placental land mammal aside from murids and bats to have made the water crossings to reach Australia prior to European arrival. It is thought that they arrived as a commensal animal with people, some time in the mid Holocene. However, the timing of their arrival is still a subject of major debate with published age estimates varying widely. This is largely because the age estimates for dingo arrival are based on archaeological deposit dates and genetic divergence estimates, rather than on the dingo bones themselves. Currently, estimates vary from between 5000–4000 years ago, for finds from archaeological contexts, and as much as 18,000 based on DNA age estimates. The timing of dingo arrival is important as post arrival they transformed Indigenous societies across mainland Australia and have been implicated in the extinction of a number of animals including the Tasmanian tiger. Here we present the results of direct dating of dingo bones from their oldest known archaeological context, Madura Cave on the Nullarbor Plain. These dates demonstrate that dingoes were in southern Australia by between 3348 and 3081 years ago. We suggest that following their introduction the dingo may have spread extremely rapidly throughout mainland Australia.

## Introduction

Aboriginal people reached Australia perhaps as much as 65,000 years ago^1^ but dingoes do not appear in the fossil record until the mid to late Holocene. As no land bridge connected Australia to Southeast Asia, and the minimum distance between Australia and Southeast Asia by island hopping during the Holocene is in the order of 100 km, the arrival of dingoes without humans is unlikely. Dingoes remain the only hard evidence for external visits by people to mainland Australia after first Indigenous settlement until about 400 years ago, when European trading ships sailed up the western coast to the East Indies and Macassan trepangers visited the northern shores^2^.

The exact time of the arrival of the dingo is somewhat equivocal with some mtDNA research suggesting as much as 18,000 years ago^3^ and, according to Savolainen *et al*.^4^, about 5000 years ago (see Fillios and Taçon^5^ for recent review). There are few published radiometric dates that directly date dingo remains and most are less than about 1000 years (Table S1^6^). The one exception to this is a date obtained from desiccated flesh from a dingo in Thylacine Hole, a palaeontological site on the Nullarbor Plain that returned the date NSW30 2200 ± 96 BP (2381–1931 cal. BP at 94.9%)^7^.

**Figure 1:**
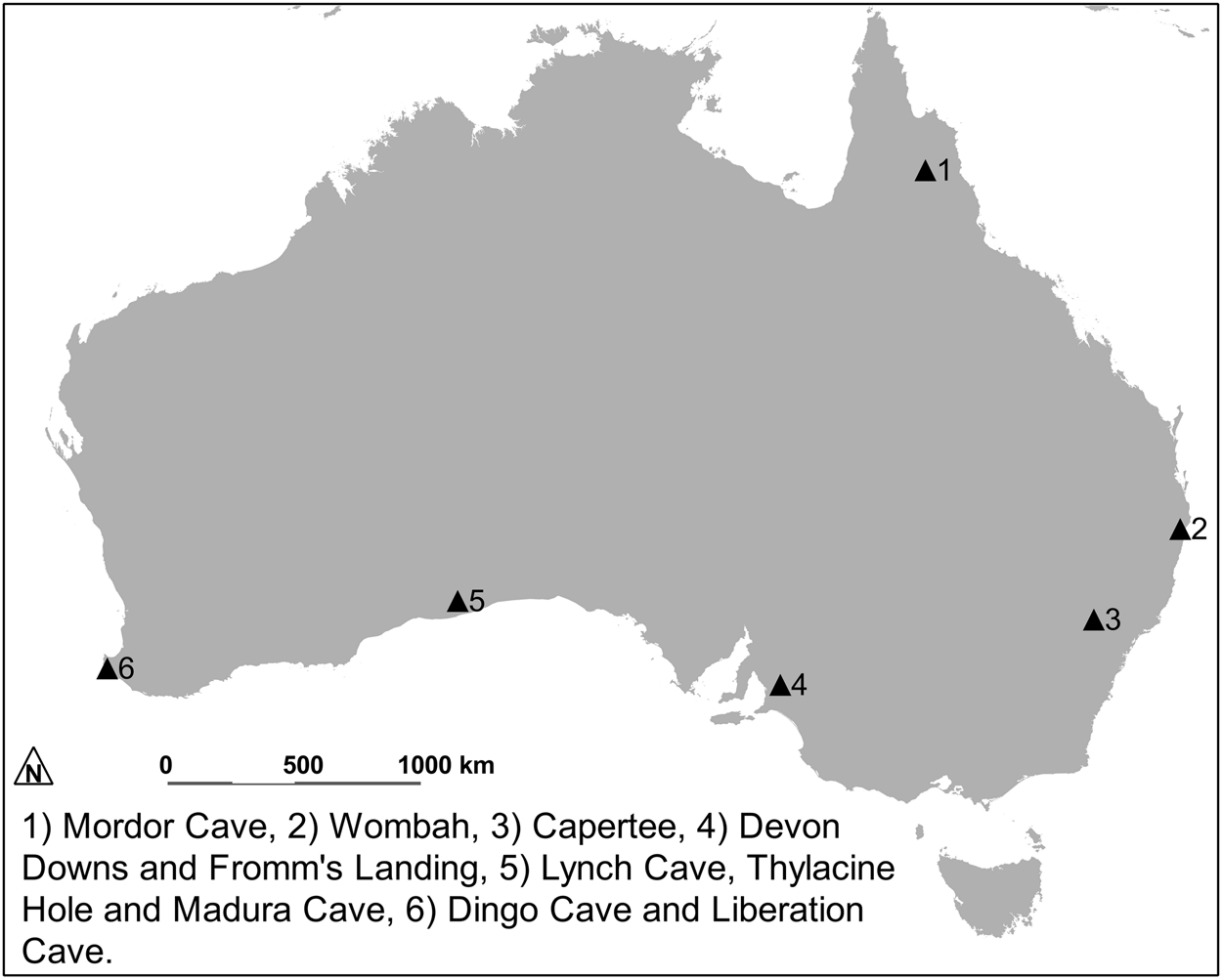
Australia with the location of places mentioned in the text.

However, the general perception and the most commonly quoted date for the arrival of the dingo in Australia is 5000–4000 years ago^8^. The four earliest radiocarbon dates in presumed good stratigraphic association with dingo remains provide calibrated dates of between 3,000 and 4,000 years. The remains derive from Capertee 3 with the date V-33, 2865 ± 57 (3080–2781 cal. BP at 93.2%)^9^, Fromm’s Landing (Tungawa) from sediments between two dates NPL89, 3170 ± 94 (3567–3076 cal. BP at 95.4%) and NPL88, 2950 ± 91 (3339–2802 cal. BP at 95.4%)^10^, Wombah midden Gak568, 3230 ± 100 (3645–3158 cal. BP at 94.6%)^11^ and Madura Cave ANU807, 3450 ± 95 BP (3905–3446 cal. BP at 94.8%)^12^. Madura Cave, from where the oldest date derives, is on the Nullarbor Plain at the southern edge of Australia (Fig. 1) and this fact, along with Gollan’s proposal^13^ that it would take no more than about 500 years for dingoes to reach the Nullarbor Plain from their presumed northern entry point, has led to the commonly quoted arrival date.

Leaving aside the question of the time taken for dingoes to colonise the continent, there remains uncertainty about the association between the Madura Cave date and the dingo remains. Two trenches separated by a one metre baulk were excavated in the site by Marun in 1969^14^: Trench 6, a one by three metre rectangle and Trench 8, a one by two metre rectangle. Although Marun does not provide stratigraphic sections, he provides detailed descriptions of the stratigraphy^14^. There were some slight stratigraphic differences between the two trenches but Marun identified five main stratigraphic layers in the excavations. All of the dingo bones were obtained from Layer 2^12^ that begins 10–20 cm below the surface to a depth of about 195 cm below the surface^14^. A series of three radiocarbon dates obtained from a single trench - Trench 8^14^ were obtained from charcoal and are in chronological order. Two of the dates are from Layer 2, one (presumed to be associated with the oldest dingo), was collected from 90 cm below the surface^14^ and the second from the base of Layer 2 at 195 cm provided a date of ANU800 4260 ± 130 (5276–4419 cal. BP). Charcoal from 330 cm below surface Layer 3 provided a date of ANU396 7880 ± 390 (7876–9632 cal. BP)^14^. This evidence, together with the stratigraphic descriptions provided by Marun^14^ that record distinct differences between each layer, suggest that there is little mixing in the deposit below the top 10 cm (Layer 1). Despite this, the association of the claimed oldest dingo bones and the dated charcoal requires verification. First the deepest dingo bones were from the 75–90 cm unit in trench 6, not trench 8, where the dates were obtained and where the deepest dingo bones were recovered from spit 4, 45–60 cm below the surface^14^. Second, the site was excavated in 15 cm excavation units so it is not possible to know where, within each 15 cm excavation unit, the bones were found.

Similar problems affect other indirect dates for dingoes collected from sites excavated before this century after which smaller excavation units became the norm and radiocarbon dates became cheaper and more accessible. Of the 15 subfossil dingo dates on the *FosSahul* database^15^ only three were considered reliable using their stringent criteria that assessed the reliability of the dating methods used and the quality of the stratigraphic relationship between the date and the dingo remains^16^. One of these dates is the direct date from Thylacine Hole discussed above and the remaining two are indirect dates on charcoal that has been assessed as reliably associated with dingo remains from Mordor Cave, an archaeological site in north east Australia (Fig. 1)^17^. All three dates are less than 2500 years old.

Accurate dating of the dingo is important for identifying the effects of its introduction. In addition to their impacts on Aboriginal societies as a commensal^18^, dingoes have been hypothesized to be implicated in the extirpation of the Tasmanian tiger or thylacine (*Thylacinus cynocephalus*) and Tasmanian devil (*Sarcophilus* sp.) in mainland Australia^19,20^. The mechanism and precise role of the dingo in these extirpations has been debated; whether for example, dingoes reduced the amount of carrion available for devils^20^, out-competed the thylacine^21,22^ or even reduced their populations by direct killing^23^.

Conversely, other researchers have argued that mid to late Holocene climate change, in particular instability associated with the onset of El Niño–Southern Oscillation (ENSO), changes in people’s technology, human population increases or a combination of these factors, sometimes coupled with the introduction of the dingo, contributed to the extirpation of one or both of these species on the Australian mainland^8,24–26^.

Arguments that the dingo was a prime mover in the extirpation of these species in mainland Australia have rested on the assumption that the disappearance of thylacine and Tasmanian devil post-dated the arrival of dingoes, and on negative evidence that in Tasmania, which the dingo did not get to, the thylacine survived into the European contact period and the devil is found to this day. However, the lack of many direct dates for these three species has made it difficult to assess the relationships between the timing of the dingo’s arrival and the disappearance of the thylacine and devil. Greater numbers of direct dates on thylacine and devil subfossils has improved the reliability of these dates. White *et al*.^27^ obtained 20 thylacine and 24 devil direct radiocarbon dates including the youngest mainland dates for each, 3290 ± 49 cal. BP and 3245 ± 62 cal. BP respectively^27^. Using these new direct dates as well as dates for these species from the *FosSahul* database that had been assessed by Rodríguez-Rey *et al*.^15^ as reliable, White *et al*.^27^ modeled the likely mainland extirpation time for the two species as being 3227 years BP (CI:3170–3281) for thylacines and 3179 years BP (95% CI:3131–3224) for devils, suggesting a synchronous extinction. As these dates are older than the oldest reliable dingo dates in the *FosSahul*, direct dates of the same order of reliability are needed to investigate whether or not dingoes did indeed arrive a thousand years before the extirpation of thylacines and devils.

In this paper we present the results of direct dating of dingo bones from Madura Cave – also known as Mereguda Cave^14^ on the Nullarbor Plain, the oldest subfossil context from which they are known, and discuss the likely rate of dingo spread from their point of arrival, throughout Australia.

## Results

We selected two samples of dingo bone for direct AMS dating from the Madura Cave dingo remains, one from spit 6 (75–90 cm) in Square I6 and one from spit 4 (45–60 cm) in Square H8, representing the deepest dingo bones from each trench. Both of the dated samples were derived from the distal phalanges.

**Table 1:** Direct radiocarbon dates obtained from dingo bone recovered from Madura Cave.

Lab. ID	Sample square/spit number	Date (BP)	%C	C:N	δ^13^C	δ^15^N	% collagen yield	Calibrated age (BP)
SANU-54820	H8/4	2105 ± 25	42.6	3.3	−16.7	11.4	4.4	2143–1932 (95.4%)
SANU-54821	I6/6	3069 ± 27	42.6	3.1	−17.9	14.9	1.3	3348–3081 (95.3%)

The results are presented in Table 1. Collagen quality in the samples was good, with more than 5 mg and 1 wt% collagen recovered from both samples. %C, C:N ratio are representative of bone collagen^28^ and stable isotope values are as expected for arid Western Australia.

The oldest bone sample dated is from spit 6 with an age estimate of 3363–3211 cal. BP (3069 ± 27 SANU 54821). There is no overlap between the calibrated dates for bone from spits 4 and 6, the smallest possible gap between the dates is 115 years and the largest is 720 years indicating that the dates are on two separate individual dingoes deposited in the site at different times, rather than one individual distributed over several spits.

## Discussion

The new dates from Madura Cave provide further evidence for the lack of site disturbance and the direct date from spit 6 in Madura Cave of about 3250 cal. BP is currently the oldest reliable date for the dingo in Australia. In our view, this date is very likely close to the time of first arrival of this species. The common reference in the archaeological literature to the dingo’s arrival as between 5000 and 4000 years ago is based on an estimate of the time it might have taken for dingoes to reach Madura Cave from northern Australia using the previous indirect date of about 3500 BP. Even the suggested date of 5000 BP, based on assumptions of mutation rates by Savolainen *et al*.^4^, is supported by referring to the indirect Madura Cave date^4^. Thus, all of the estimates of a mid Holocene arrival date for dingoes in Australia have assumed that colonization across the continent took between 500 to 1500 years.

We see no reason to suggest such a long colonization time. Instead, we suggest that dingo dispersal from north to south may have been accomplished within the 95% probability range of the calibrated radiocarbon date for SANU-54821. As the presence of dingoes on watercraft indicates that they were tamed animals when they arrived in Australia, it is likely that Aboriginal people took them up very rapidly and that this association facilitated their movement across the continent. Some idea of the potential rapidity of the commensal relationship and how this may have assisted dingo spread may be gauged by the uptake of the dog by Indigenous Tasmanians when it was introduced across the Tasman at the time of European contact. Both Jones^29^ and Boyce^30^ summarise the historical references to early dog adoption by Tasmanian Aboriginal people. Introduced in 1804 by Europeans to help hunt kangaroos^30^, they were quickly acquired by Aboriginal people. Jones^29^ mapped the distribution of Robinson’s observations of dogs during his journeys 1829–1835. He notes that in 1830, when Robinson arrived on the west coast of Tasmania, European contact had only been ongoing for five to eight years and that, although Robinson was the first white man the local Aboriginal people had met at close quarters, they had already acquired dogs.

The length of time for the dispersal of cats across the Australian continent also provides a good comparison because, like the dingo, it is a commensal and people assisted its movement. Abbott^31,32^ analysed numerous historical documents to identify the distribution of cats within Australia. He found that there were multiple introductions as they settled various regions of Australia becoming feral in Sydney in 1820, in Perth and south-east Australia by 1840 and over 90% of the continent by 1890. While there may have been more than one entry point, cats were present in central Australia by the late 1800s, suggesting that whatever their entry point, their dispersal across the continent took a mere 70 years. As Aboriginal people occupied that whole continent when dingoes arrived, they no doubt assisted swift movement by providing a network through which dingoes moved. Thus, we believe, that the dispersal of dingoes could have been just as rapid as cats.

The new Madura Cave date fits well with dates for dogs in mainland and island Southeast Asia. Piper’s^33^ thorough review of the distribution and age of dogs in archaeological contexts in these regions found that there is no reliable evidence for dogs in mainland Southeast Asia prior to ca. 4000 cal. BP when it is found in both north and south Vietnam. The oldest reliable date for dog in island Southeast Asia is from Timor-Leste, where a direct date on a dog burial produced an age of 3000 cal. BP^34^. It is worth mentioning that older dates for dog from Timor-Leste, that are often referred to in published literature, are based on indirect associations with radiometric dates and are not reliable. These dates are sometimes suggested to be 5000 years^35^ and sometimes 3500^4^. However, Glover, who excavated the sites in question, suggested that the oldest reliably identified dogs were from Bui Cero Uato, which, in the absence of radiocarbon dates, Glover correlates with the artefact sequence and concludes that they are dated to between 3500 and 2500 BP^36^.

The new Madura Cave date is only slightly older than the dates obtained for the most recent thylacine and devil remains in mainland Australia modeled by White *et al*.^27^ to be between 3227 and 3179 years ago, and it might be tempting to see a causal relationship in this correlation. However, records of El Niño-Southern Oscillation (ENSO) strength indicate changes occurred in the mid to late Holocene that very likely had a dramatic impact on thylacine and devil habitat and prey species numbers. Proxy climate records for mainland Australia indicate a transition from a tropical humid climate with predictable rainfall in the early to mid Holocene, to a much drier climate where the summer monsoon was absent or intermittent, beginning about 5000 years ago and intensifying, which was sustained until about a thousand years ago or later^37–46^. Australian archaeologists have noted this as a time of major change in stone tool assemblages with the addition of new stone tools used in hunting and innovative methods of reduction which they link to the need to offset foraging risk first appearing at this time^47–52^. For example, Maloney *et al*.^51^ argue that although points first appear in Kimberley sites at about 5000 cal. BP, their production intensifies after about 3600 cal. BP as a strategy to offset foraging risk. Extinction events rarely have a single cause. The survival of both species in Tasmania may have had as much to do with higher rainfall in the mid to late Holocene in this region as it did with the lack of competition from dingoes.

## Methods

### Sample selection

Five one metre squares were excavated at Madura Cave in 15 cm spits^16^. The deepest dingo bones were recovered from Trench 6, Square I, spit 6 (75–90 cm below surface) and from spit 4 (45–60 cm) in Trench 8, Squares H and I. These bones were selected as, if as discussed above the site is relatively undisturbed, they should represent the oldest dingo remains in each trench. Further, as the bone from spit 4 were recovered from above the charcoal date previously suggested to provide a date for dingoes at the site, they should produce a younger date, whereas the bone from Trench 6 should be closer to the original date.

### Dating

Collagen was extracted and purified from the dingo bones following an ultrafiltration protocol described by Wood *et al*.^53^. Briefly, bone powder was demineralized in 0.5 M HCl overnight, and then washed in 0.1 M NaOH (30 minutes) and 0.5 M HCl (1 hour), rinsing between treatments with ultrapure water. Samples were gelatinized in 0.001 M HCl (70 °C, 20 hours), filtered with an Ezee-Filter^TM^ to remove insoluble contaminants and ultrafiltered with a cleaned Vivaspin^TM^ VS15turbo 30 kDa MWCO ultrafilter to remove small contaminants. For dating, freeze-dried collagen was combusted in a sealed tube with CuO wire and Ag foil, and the CO_2_ generated cryogenically purified and collected for graphitization with H_2_ over an Fe catalyst, and analysis in a NEC single stage AMS at the Australian National University^54^. Dates were calculated according to Stuiver and Polach^55^ and a bone specific background correction made. Dates have been calibrated SHCal13^56^ in OxCal v.4.3^57^ and 95.4% probability ranges are given for calibrated dates unless otherwise stated. To assess collagen quality, carbon and nitrogen elemental and stable isotope abundances were obtained from a second aliquot of collagen using an ANCA GSL connected to a Sercon 20–22 IRMS operating in continuous flow mode, using an in-house gelatin reference and corrected against USGS40 and USGS41.

## Conclusion

The oldest direct date for dingoes in Australia is 3348–3081 cal. BP from Madura Cave on the Nullarbor Plain in the south of the continent. The origin point of the dingo must be from the north of Australia and thus it has been thought that the true arrival date must be earlier than this. We have argued that the dispersal of dingoes is likely to have occurred very rapidly and possibly within the age range of the calibrated date, especially as humans probably facilitated their spread. We have previously argued^18^ that dingoes would have quickly formed a commensal relationship with Aboriginal people and would have changed subsistence, gender relationships and other aspects of Indigenous societies. The dispersal of dingoes has been also associated with major changes in the Australian biota as a result of hunting pressure, and in the case of the thylacine and devil, competition. Determining whether or not dingoes had a role in these changes requires precision in dating the arrival of dingo and extirpation of the thylacine and devil in mainland Australia. Recent direct dating and modeling has achieved this for thylacine and devil extirpation but reliable dates for dingo introduction have been lacking. We have made a start on this by dating the dingo remains from the oldest archaeological context currently known in Australia. Further direct dating on dingo bones from northern Australian contexts is now required to test our hypothesis for rapid dingo spread.
